# Real-time assessment of corneal endothelial cell damage following graft preparation and donor insertion for DMEK

**DOI:** 10.1371/journal.pone.0184824

**Published:** 2017-10-04

**Authors:** Maninder Bhogal, Chan N. Lwin, Xin-Yi Seah, Elavazhagan Murugan, Khadijah Adnan, Shu-Jun Lin, Gary Peh, Jodhbir S. Mehta

**Affiliations:** 1 Singapore Eye Research Institute, Singapore, Singapore; 2 Singapore National Eye Centre, Singapore, Singapore; 3 Institute of Ophthalmology, University College London, London, United Kingdom; 4 Duke-NUS Graduate Medical School, Singapore, Singapore; Cedars-Sinai Medical Center, UNITED STATES

## Abstract

**Purpose:**

To establish a method for assessing graft viability, in-vivo, following corneal transplantation.

**Methods:**

Optimization of calcein AM fluorescence and toxicity assessment was performed in cultured human corneal endothelial cells and ex-vivo corneal tissue. Descemet membrane endothelial keratoplasty grafts were incubated with calcein AM and imaged pre and post preparation, and in-situ after insertion and unfolding in a pig eye model. Global, macroscopic images of the entire graft and individual cell resolution could be attained by altering the magnification of a clinical confocal scanning laser microscope. Patterns of cell loss observed in situ were compared to those seen using standard ex-vivo techniques.

**Results:**

Calcein AM showed a positive dose-fluorescence relationship. A dose of 2.67μmol was sufficient to allow clear discrimination between viable and non-viable areas (sensitivity of 96.6% with a specificity of 96.1%) and was not toxic to cultured endothelial cells or ex-vivo corneal tissue. Patterns of cell loss seen in-situ closely matched those seen on ex-vivo assessment with fluorescence viability imaging, trypan blue/alizarin red staining or scanning electron microscopy. Iatrogenic graft damage from preparation and insertion varied between 7–35% and incarceration of the graft tissue within surgical wounds was identified as a significant cause of endothelial damage.

**Conclusions:**

In-situ graft viability assessment using clinical imaging devices provides comparable information to ex-vivo methods. This method shows high sensitivity and specificity, is non-toxic and can be used to evaluate immediate cell viability in new grafting techniques in-vivo.

## Introduction

Corneal disease ranks second only to cataract as the leading cause of preventable blindness[1], and corneal transplantation is the commonest tissue transplant procedure performed worldwide. Whilst scarring and infection are major causes of corneal sight-loss in the developing world, in the USA, Europe and parts of Asia (e.g. Singapore and Australia) endothelial dysfunction is the primary indication for corneal transplantation[2]. The cornea’s transparency is dependent upon the function of the non-replicative corneal endothelium, which pumps water out of the corneal stroma, maintaining corneal hydration and permitting optimal light transmission[3]. If the endothelial cell density (ECD) falls below approximately 500 cells/mm^2^, failure of this pump layer occurs, resulting in edema, opacity and ultimately scarring and vascularization if left untreated[4]. Currently the only mechanism to replace damaged endothelial cells is transplantation.

Owing to the immunological privilege of the eye, the early success rate for penetrating keratoplasty is very high[5]. However, ongoing endothelial cell loss occurs in the transplanted tissue, and proceeds at an accelerated rate compared to that observed in the native corneal endothelium of healthy adults (4.2% for penetrating keratoplasty vs 0.3% in the native corneal endothelium) [6,7]. This means that late graft failure is a common occurrence[8] requiring re-grafting, which in itself is a common indication for keratoplasty [2]. Over the past 2 decades, endothelial keratoplasty (EK), in which the patients’ diseased endothelium is selectively replaced with donor endothelial cells, has surpassed penetrating keratolasty in developed nations[2]. However, there is concern that these more technically demanding procedures, may result in a lower number of endothelial cells being transplanted and may, therefore, have reduced survival times[9].

Pooled data from large patient series consistently show a reduction in endothelial cell density in the early post-operative period[7,10,11]. Damage to the donor cornea may occur following the initial death of the donor, harvesting of the cornea, storage of the tissue in culture media[12], graft preparation[13,14], graft insertion[15,16] and finally from manipulation within the anterior chamber.

Reducing cell loss relies upon accurate methods to assess cell viability at each stage of the transplantation process, allowing the cause at each point to be targeted for refinement. Current ex-vivo methods include staining with alizarin red S & trypan blue[17], scanning electron microscopy[16,18] and the use of fluorescence based viability dyes[19] or apoptosis assays[20], many of which are incompatible with subsequent transplantation and in-vivo assessment. An ideal assessment tool would be one in which viability can be determined at a single cell level across the entire graft[13] and performed sequentially within the same sample.

Gauthier et al performed a study comparing early post-operative cell loss following PK to whole graft viability performed in the fellow cornea from the same donor[21]. In a minimally traumatic procedure such as PK, no difference was found in early post-op cell density and that calculated after adjusting for whole graft viability. However, a significant difference between raw pre- and post-operative central cell density was found.

In EK, studies have shown graft preparation only accounts for a small percentage of the cell loss typically observed in the early post-operative period, suggesting proportionally more cells are lost from the surgical process itself[13,22]. Determination of the cell loss attributable to surgery has, until now, relied upon surgical models designed to simulate the surgery. Some of these bear poor resemblance to the surgery itself[23] and where animal models have been used, there is a need to sacrifice the animal in order to perform detailed analysis of the impact of surgery on cell survival[24]. Currently no method for immediate, detailed, in-vivo, post-operative viability assessment exists.

In this paper we present a method that allows accurate, in-situ assessment of graft viability after a single incubation of a fluorescent viability dye prior to tissue preparation. We show that changes in viability can be tracked through all stages of graft preparation and implantation using a commercially available clinical confocal scanning laser microscope. With only minor adaptation to standard setup, viability could be determined in-vivo both globally and at the single cell level.

## Methods

### Corneal tissue and preparation

Approval for this study was granted by the Singhealth centralized institutional review board. Experimentations were conducted in accordance with the tenets of the Declaration of Hensinki. Human corneo-scleral buttons with consent for research use were obtained from Miracles in Sight (Winson Salem, North Carolina, USA) and Lions Eye Institute for Transplant and Research (Tampa, FL, USA). Ex-vivo tissue preparation and experimentation was performed by a single surgeon (MB) with experience of >200 DMEK procedures, where transplant grade tissue with endothelial cell counts of >2200 cells/mm^2^ and a storage time <14 days in Optisol GS (Bausch & Lomb Rochester, New York) was used. Studies involving the isolation and expansion of primary human corneal endothelial cells for in-vitro toxicity and flow cytometry studies uses research grade tissue with endothelial cell counts of >2000 cells/mm^2^ with a similar storage time of <14 days in Optisol GS. Detailed tissue information is available in Table 1.

**Table 1:** Donor information.

Serial Number	Age (Years)	Sex	Ethnicity	Time of Death to Preservation	Storage Time (Days)	Cell Count per mm^2^	Cause of Death	Experiments Performed
1	44	F	Black	4H 2M	11	2671	Respiratory Disease	In-Situ Imaging SEM
2	68	M	White	13H 15M	12	3150	Respiratory Disease	
3	63	F	White	1H 45M	10	3035	Respiratory Disease	
4	63	F	White	12H 16M	11	2903	Respiratory Disease	In-Situ Imaging Alizarin Red & Trypan Blue Staining
5	62	M	White	12H 44M	11	1681	Heart Disease	
6	21	M	Black	2H 47M	13	3178	Trauma	
7	58	F	White	8H 31M	12	2774	Multi-Organ Failure	In-Situ Imaging Annexin V Assay
8	52	M	White	6H 4M	14	2807	Cancer	
9	51	M	White	20H 12M	11	2507	Liver Failure	In-Situ Imaging
10	58	M	White	15H 10M	11	3408	CVA	
11	46	F	White	19H 43M	12	2778	Sepsis	
12	64	M	White	5H 34M	12	2364	Respiratory Disease	
13	65	F	White	8H 0M	9	2329	Cancer	
14						2215		
15	57	F	White	16H 55M	12	2477	Heart Disease	
16	42	M	Black	14H 36M	14	2916	Trauma	
17	67	M	Black	6H 51M	10	2824	Sudden Cardiac Death	
18	50	M	White	14H 50M	14	3078	Heart Disease	Ex-Vivo Toxicity
19						3186		
20	16	M	White	12H 19M	11	3212	Sudden Cardiac Death	
21						3306		
22	58	M	Black	15H 52M	9	2003	Heart Disease	
23	64	M	White	5H 40M	12	2343	Respiratory Disease	
24	59	M	White	6H 25M	13	2731	Cancer	
25	58	M	Asian	6H 54M	13	3360	End Stage Renal Disease	
26	64	M	White	8H 9M	14	2801	Heart Disease	Annexin V Assay Ex-Vivo Pan Corneal Imaging
27	65	F	White	19H 24M	11	3154	Cancer	
28	59	M	White	6H 16M	11	2335	Renal Failure	
29	44	F	White	1H 23M	12	3015	CVA	
30	58	F	White	17H 36M	12	2066	Heart Disease	In-Vitro Toxicity
31	55	M	White	23H 3M	12	2628	Heart Disease	
32						2946		
33	51	M	White	6H 47M	12	2813	Liver Failure	
34	24	M	White	12H 18M	9	3333	Multiple Blunt Trauma	
35						3185		
36	23	M	White	18H 16M	10	3125	Multi-Vehicle Accident	
37						3058		
38	21	F	White	4H 42M	7	3401	Renal Failure	
39						3195		
40	29	F	Black	21H 7M	6	2907	Cholangiocarcinoma	
41						3311		
42	13	M	White	18H 59M	8	2865	SI-GSW-Head	
43						2950		
44	21	M	White	16H 41M	8	3205	Bowel Obstruction	
45						3584		
46	17	F	White	21H 50M	12	3472	Hanging	In-Vitro Toxicity Flow Cytometry
47						3571		
48	28	M	Black	25H 26M	6	3268	Head Trauma	Flow Cytometry
49						3236		
50	18	M	Black	23H 20M	6	3268	Subarachnoid Hemorrhage	
51						3257		

A total of 51 donor corneas, of which 13 were pairs, were used in this study. Donor age ranged from 13 year-old to 68 year-old with a median age of 52 year old. Days taken from death of donor to the usage of the donor tissues ranged from 6 days to 14 days with a median of 11 days.

We have previously described our preparation method for DMEK transplants in detail[13]. In brief, manual peeling was performed on the punch block of a standard trephine (Coronet, Network Medical, UK). After 360° peripheral scoring with a Sinsky hook, the DM/endothelium complex was peeled 90% off, laid back flat and punched with an 8mm trephine. A triangular orientation mark was made in the periphery of the graft[25] before it was gently washed with BSS and returned to the viewing chamber for imaging.

### Cell culture

Human corneal endothelial cells (HCECs) were isolated using a two-step ‘peel and digest’ method as described previously[26]. Briefly, isolated HCEC’s were first established in cornea endothelial maintenance/stabilization medium (M5-Endo) consisting of Human Endothelial-SFM supplemented with 5% Fetal Bovine Serum (FBS) (Life Technologies, Carlsbad, CA, USA) overnight. Subsequently, HCECs were cultured in a proliferative medium (M4-F99) consisting of Ham's F12/M199, 5% FBS, 20 μg/ml ascorbic acid, 1× insulin/transferrin/selenium (Life Technologies) and 10 ng/ml basic fibroblast growth factor (R&D Systems, Minneapolis, MN, USA) to promote the culture of HCECs. Once HCECs became 80%–90% confluent, M5-Endo was re-introduced and cells cultured for at least two days before being passaged via trysin-EDTA dissociation. Dissociated cells were plated at a seeding density of at least 1 × 10^4^ cells/cm^2^ in culture dishes coated with a cell attachment reagent (FNC coating mix, United States Biologicals Swampscott, MA, USA) for subsequent cellular expansion. All cultures were incubated in a humidified atmosphere at 37°C and 5% CO_2_. Cells were collected after at least 2 rounds of passaging using the above expansion protocol and seeded at a density of 2500 cells/mm^2^ for all in-vitro experimentation.

### Fluorescence optimization

HCECs were seeded in a Greiner 96-well flat bottom polystyrol microplate (Greiner, Frickenhausen, Germany) pre-coated with FNC coating mix. Cells were incubated with varying concentrations of calcein AM (2–8 μM) for 30 mins at 37°C as per the manufacturer’s instructions (n = 8 per concentration). Cells were washed x3 in fresh culture media. Fluorescence intensity was measured at 540 nm using the Tecan multimode plate (Tecan infinite M200 pro, Zanker Road, San Jose, USA) reader set at an excitation frequency of 488nm. Fluorescence was measured at hourly intervals for the first 4 hours and then at varying intervals up to 96hrs. Once calcein AM concentration had been optimized in HCECs, staining was performed in whole corneas. Sequential imaging was performed in corneas stored in organ culture (37°C) (n = 2) or Optisol (4°C) (n = 2) for 1–14 days.

### Calcein AM toxicity assessment

Toxicity assessment was conducted in HCECs and organ cultured human tissue.

### Flow cytometric assessment

To determine if calcein AM had any inherent toxicity in primary cultured HCECs, a propidium iodide (PI) exclusion assay was used. Cell cultures were exposed to calcein AM (2–8μM) for 30 mins prior to washing and trypsinisation. Suspended cells were incubated in a solution containing PI in the dark for 15 minutes, following the manufacturers instructions, (BioLegend, San Diego, CA, USA), before being analyzed using a FACS Verse flow cytometer (Becton Dickinson, East Rutherford, NJ, USA) to determine the percentage of PI positive (dead) cells.

### Tissue culture assessment

Two, full-thickness, 5mm discs where punched from each transplant grade cornea. One disc was incubated with calcein AM (2.67μM) for 30 minutes, washed 3 times in fresh culture media and incubated for a further 48 hrs at 37°C. The control disc was incubated in culture media without calcein AM and washed in the same manner. After incubation, corneal discs were triple stained with calcein AM (2μM), ethidium homodimer (4μM) and Hoeschst 33323(10μM). Samples were coated with an ophthalmic viscoelastic (Viscoat, Alcon, Forth Worth, Texas, USA) and imaged. Viability was assessed in at least 1000 cells from three non-contiguous regions free from handling/trephination damage. Percentage of dead cells, hexagonality ratio and coefficient of variation was calculated and compared between calcein AM treated cells and untreated controls.

### Surgical model of DMEK

**Fig 1:**
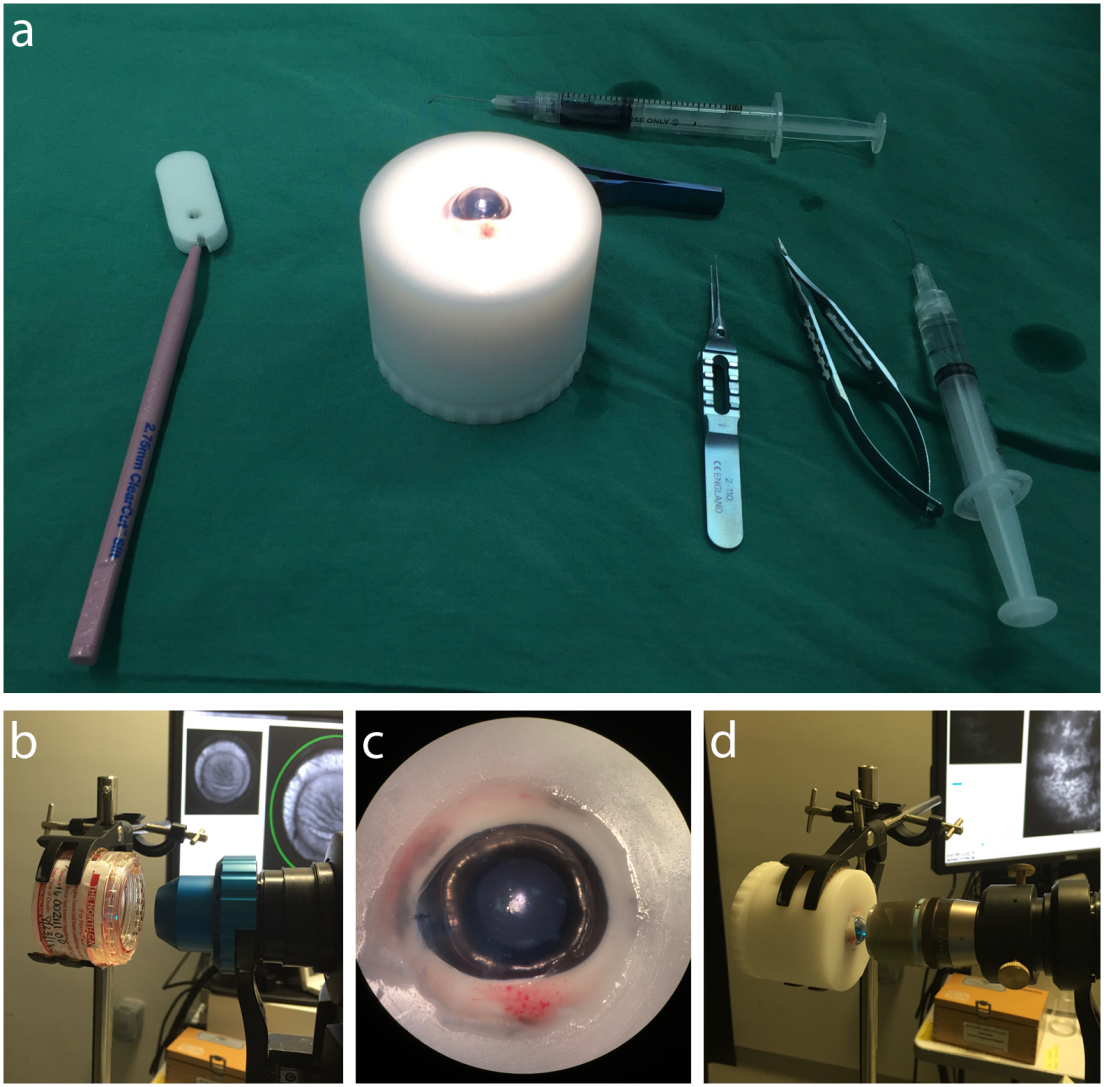
Experimental setup of DMEK surgical model used. (A) Image showing porcine eye mounted within holder and standard equipment used for DMEK surgery. (B) Tissue is imaged prior to and following DMEK preparation whilst still inside the standard Optisol viewing chamber. Use of the standard anterior segment lens supplied by the manufacturer and the 30° field-of-view imaging setting allows visualization of the entire cornea. (C) Image showing DMEK graft unfolded within the porcine anterior chamber. (D) The graft is imaged in-situ. The addition of microscope objective lens allows non-contact, individual cell imaging.

Cornea-scleral buttons were removed from the Optisol viewing chambers and placed into a 12-well culture plate. Specimens were covered with 250μl of balanced salt solution containing 2.67 μM calcein AM (1:1500 stock solution, Life Technologies Corporation) and incubated at 37°C for 30 minutes (based on the findings of our fluorescence optimization work). Grafts were rinsed in balanced salt solution (BSS), returned to the viewing chamber and stored at room temperature until needed for imaging. Fresh porcine globes (less than 6 hours after slaughter) were obtained from a local abattoir. Experimentation was conducted either immediately after delivery (n = 10) or at 24hrs post slaughter (n = 10), allowing the cornea to swell and better simulate imaging through an edematous cornea. Globes were mounted in a customized holder and BSS was injected into the vitreous as necessary to return the ocular pressure to an approximately physiological level prior to commencing surgery. DMEK grafts that had been prepared and imaged as described earlier were peeled off fully, allowed to scroll on the stroma and stained with trypan blue dye (DORC International) for 1 minute. Excess dye was washed off and the stained DMEK scroll was transferred to a petri dish filled with BSS. The graft was drawn into a dedicated DMEK insertion device (Geuder AG, Heidelberg, Germany) and delivered into the porcine anterior chamber through a 2.8mm clear corneal incision. The grafts were unfolded using a standard ‘no touch’ technique[27] and the entire anterior chamber filled with air prior to repeat imaging of the graft in-situ. (Fig 1a)

### Imaging of cell viability in-situ

Imaging was performed at three time points; prior to DMEK preparation, following DMEK preparation and in-situ imaging of unfolded DMEK graft (Fig 1b–1d). All imaging was performed using the Spectralis^™^ HRA confocal scanning laser ophthalmoscope (Heidelberg Engineering, Heidelberg, Germany). Caclein AM fluorescence was detected using the 488 nm solid-state excitation laser and 500 nm long-pass filter. The manufacturer-supplied anterior chamber lens was used to acquire 30–50 frames, at a fixed sensitivity of 90. These were averaged using the integrated software (Heidelberg Eye Explorer, Heidelberg Engineering) to produce a single image. To increase image magnification, and allow visualization of individual cells, a x40 air interface microscope objective lens (Nikon, Tokyo, Japan) or the Rostock module(Heidelberg), a simple lens system containing a x63 water immersion lens, were coupled to the supplied 30° retinal lens using customized collars.

### Image analysis

**Fig 2:**
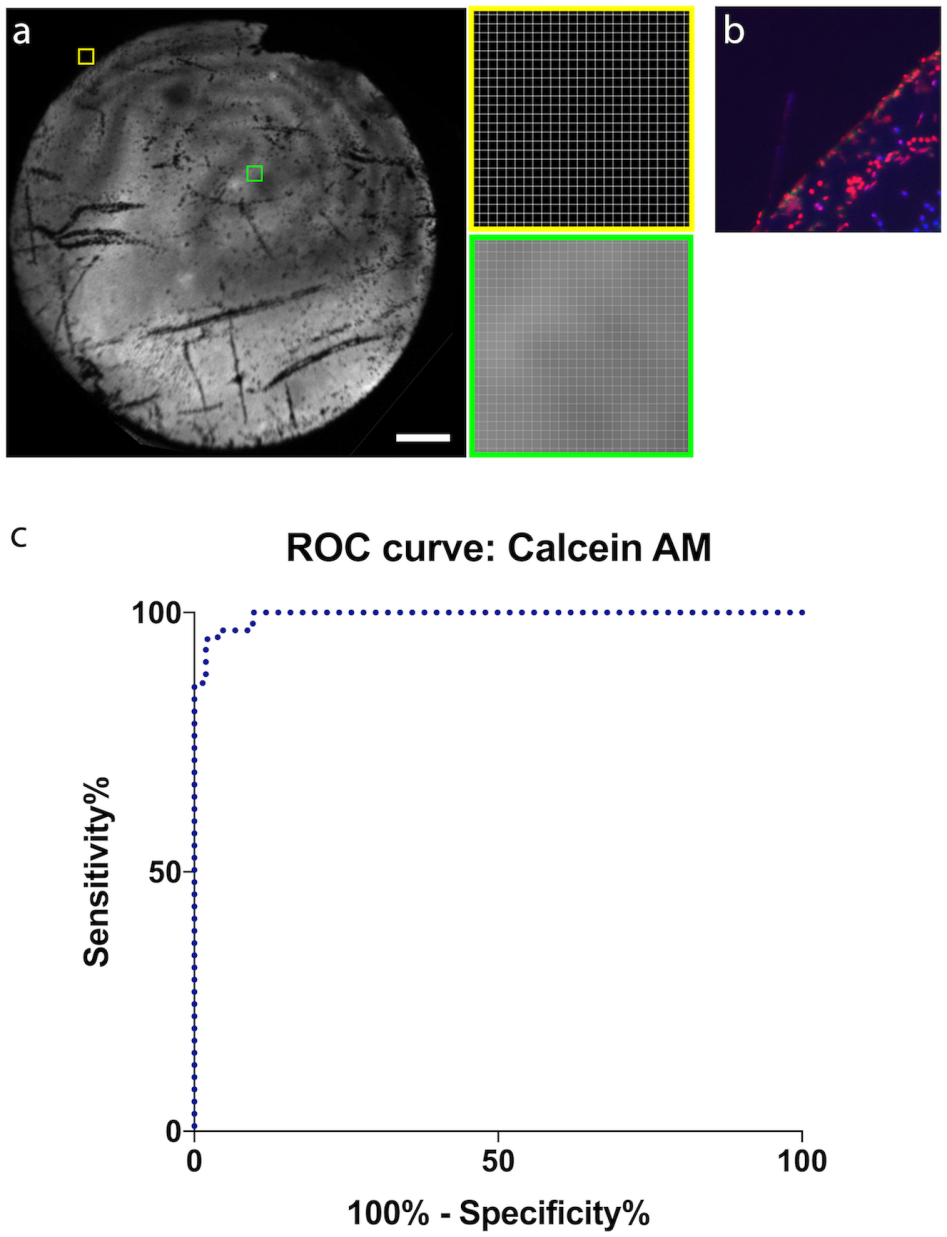
Calculation of fluorescence contrast and signal to noise ratio of the calcein AM fluorescence staining. (A) 25x25 pixel arrays in viable (green square) and non-viable areas of the graft were used to calculate fluorescence contrast and signal to noise ratio. Scale bar 1mm. (B) The periphery of the graft was chosen as a standard area of non-viable tissue as this consistently contains areas of bare Descemet membrane as well as attached, non-viable cells; stained positively with ethidium homodimer (this images corresponds to the yellow square in Fig 2A). (C) Receiver-operator-characteristic for calcein AM fluorescence.

All images were exported to Image J (National Institute for Health, Bethesda, USA) for processing and quantification. Mean fluorescence was measured from multiple 25x25 pixel arrays (Fig 2a). Signal-to-noise ratio was defined as the mean fluorescence intensity for the entire array divided by the standard deviation of fluorescence intensity (derived by measuring the intensity of each pixel within the array). Fluorescence contrast was calculated by dividing mean fluorescence of viable areas by that of adjacent non-viable areas. The periphery of the graft was chosen as a standardized area of non-viable tissue as this region is known to contain both dead cells and patches of bare Descemet membrane (Fig 2b).

To determine if calcein AM staining alone was sufficient to allow automated cell density determination, cell counting was performed on images acquired with the x40 lens array using the ITCN plugin for ImageJ. Cell density measurements generated from in-situ images were compared to those generated from the same region of the cornea by counting Hoechst positive nuclei (see later).

### In-vitro viability

Immediately after the in-situ imaging, corneas in the porcine model were excised and the DMEK grafts carefully harvested. Comparisons between our in-situ imaging method and widely used, ex-vivo methods of graft viability assessment were made.

### Fluorescent viability assessment

Calcein AM/ethidium homodimer staining is frequently used in cell biology applications to detect live/dead cells respectively. Grafts were incubated in BSS containing Calcein am (2μM) ethidium homodimer (4μM) and Hoechst 33323 (10μM) for 30 mins. Grafts were transferred to a customized curved viewing chamber and fluorescence imaged with a x4 objective lens on the Nikon TIe inverted fluorescence (Nikon) using our previously described method[13]. This allowed imaging of the entire graft, meaning a comparison between our in-situ and in-vitro methods could be conducted in the same regions of the transplant. A Bland-Altman analysis was conducted to determine if measurement of cell density derived from in-situ calcein AM fluorescence showed good agreement with that derived using nuclear counter staining with Hoechst.

### Trypan blue/Alizarin red S staining

Trypan blue/Alizarin red S viability staining is frequently used in ex-vivo graft assessment [16]. Cell borders are stained by alizarin red and dead cell nuclei stain with trypan blue. Both dyes stain and bare Descemet membrane (on both the stromal and endothelial surfaces), which can be problematic when assessing DMEK tissue as the stromal surface of the DM is exposed. The corneas were stained with 0.4% Trypan blue (Sigma-Aldrich Corp., Singapore) for 1 minute and rinsed with phosphate buffered saline (PBS, 0.01 M, Life Technologies, Carlsbad, CA, USA). The corneas were subsequently stained with 0.5% Alizarin red S (Sigma-Aldrich Corp.) for 2 minutes, and rinsed again with PBS prior to imaging on an upright light microscope with a 3-colour CCD.

### Scanning electron microscopy

Samples were fixed in glutaraldehyde 2% immediately after sacrifice. Corneas were washed twice in PBS for 10 minutes each before being immersed in 1% aqueous solution of osmium tetraoxide (FMB, Singapore) for 2 hours at room temperature. Samples were then dehydrated using increasing concentrations of ethanol (25%, 50%, 75%, 95% to 100% ethanol, with 95% and 100% concentrations being performed twice) prior to critical point dried using Bal-Tec dryer (Balzers, Liechtenstein) and mounting on stubs secured by carbon adhesive tape. They were then sputter coated with a 10-nm-thick layer of gold (Bal-Tec) and examined using the JSM-5600 scanning electron microscope (JEOL, Tokyo, Japan).

### Annexin V staining

To determine if calcein AM fluorescence could detect endothelial cells undergoing early apoptosis, 2 transplants were incubated in calcein AM blue (Excitation 322nm/Emission 435nm) and Annexin V conjugated to FITC from an apoptosis detection kit following the manufacturer’s instructions (BioLegend), San Diego, CA, USA) and imaged on the fluorescence microscope.

### Statistical analysis

Statistical analysis was conducted using GraphPad Prism 7.0 (GraphPad Software Inc, La Jolla, CA, USA). A p-value of <0.05 was deemed to be significance in all tests. Comparison between 2 groups of variables was performed using two-sided t-tests or the Wilcoxon rank test if the data was non-parametric. Where appropriate, paired sample analysis was performed. When comparing groups of three or more, analysis was performed using ANOVA.

## Results

### Optimization of calcein AM for in-vivo imaging

**Fig 3:**
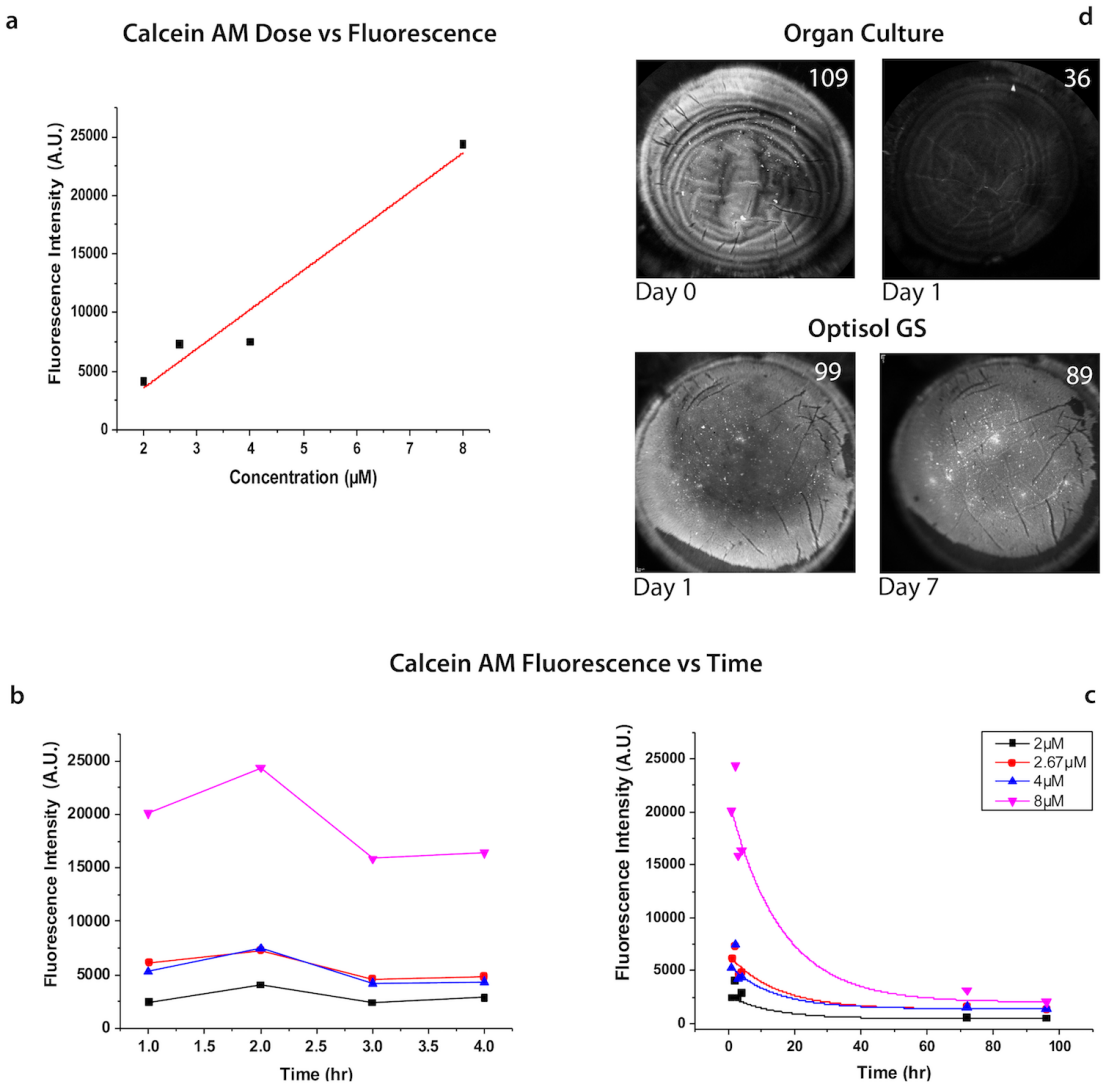
Optimization and characterization of calcein AM fluorescence for in-vivo imaging. (A) Calcein AM fluorescence shows a linear relationship with the incubation dose in cultured human corneal endothelial cells. (B) Peak fluorescence is seen at 2 hours after incubation. (C) Fluorscence dimishes rapidly over the first 24hrs in cells returned to culture. (D) Fluorescence dimishes rapidly over the first 24 hours (mean fluorescence drops from 109 to 36) in whole corneas stained with calcein AM and then returned to organ culture at 37°C, making descrimination between viable and non-viable areas not possible. For tissue stored in Optisol at 4°C, fluorescence contrast remains high at 7 days post incubation, with little change in fluorescence intensity (99 vs 89).

A linear dose:fluorescence relationship was observed for calcein AM staining in cultured endothelial cells (Fig 3a). Of the 4 concentrations tested, 2.67 μM was found to be the minimal concentration sufficient for imaging with the Spectralis^™^ HRA. This was the lowest concentration at which discrimination between viable and dead cells, both globally and at an individual cell level, could be performed reproducibly. In the majority of samples stained with 2.67μM calcein AM, nuclear staining was greater than that of the cytoplasm, thereby aiding semi-automated cell counting. As calcein AM concentration increased, the contrast between nuclear and cytoplasmic fluorescence reduced.

After incubation and washing in fresh media, fluorescence continued to peak for a further two hours (Fig 3b.), after which time point it began to rapidly decline (Fig 3c). Fluorescence was also assessed in whole corneas stored in different culture media (n = 4). By 24 hours, fluorescence dropped considerably and contrast was insufficient to discriminate between viable and non-viable regions in grafts stored in organ culture at 37°C. There was little change in mean fluorescence or contrast for grafts stored in Optisol GS at 4°C for up to 2 weeks (Fig 3d).

### Effect of CAM on in-vitro and ex-vivo cell viability

**Fig 4:**
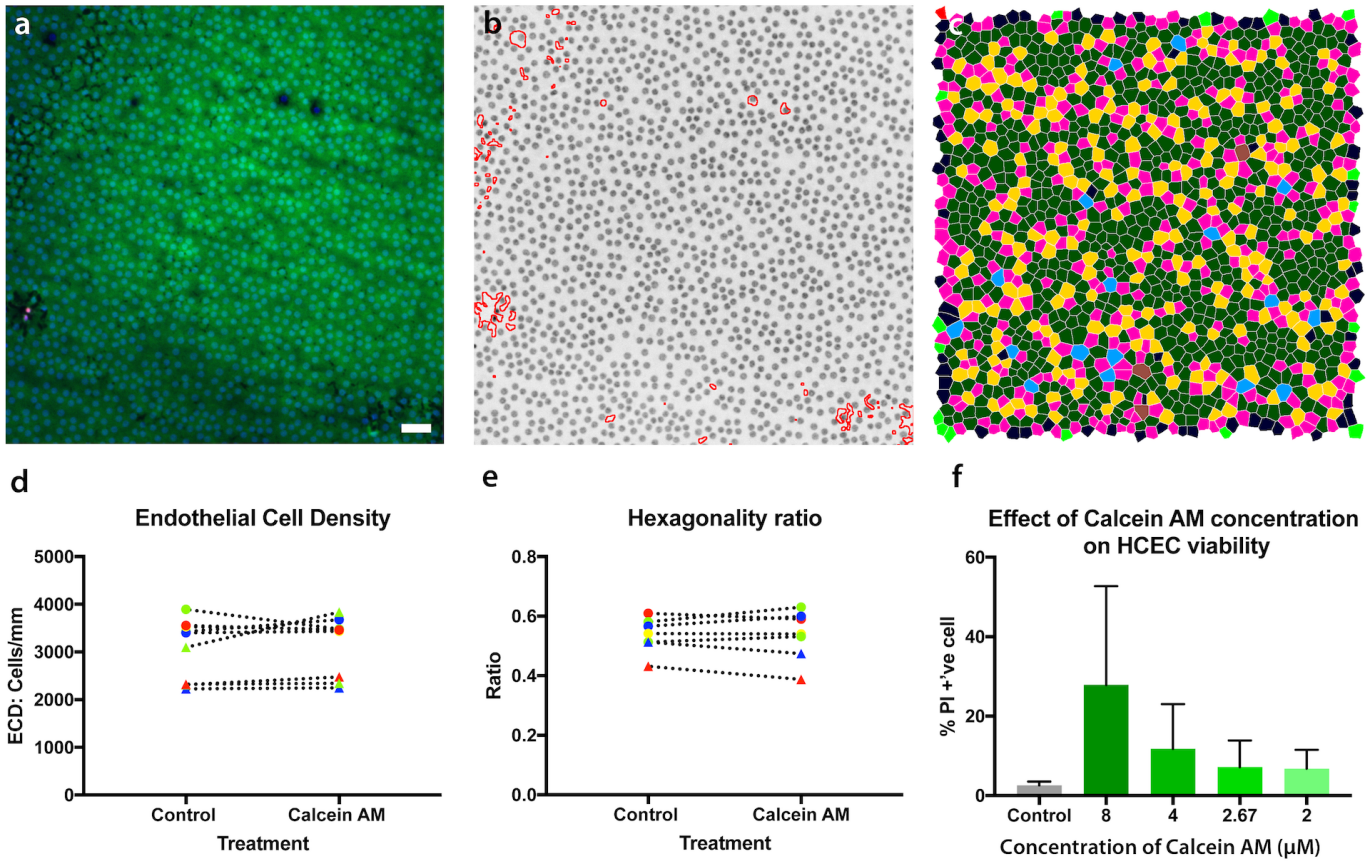
Toxicity and effect of calcein AM on ex-vivo and in-vitro cell viability. (A) Tissue triple stained in hoechst, ethidum and calcein AM. Scale bar 200μm. (B) Live cells were selected and an average live cell density calculated. (C) Cell neighbor analysis allowed the percentage of hexagonal cells (green) to be calculated. (D) Live cell density was compared in paired corneal samples (each symbol represents to unique pair of cornea). There was no significant difference between corneal endothelial viability or (E) hexagonality ratio in ex-vivo corneas pre-incubated in calcein AM and controls. (F) Flow cytometry assessment of the percentage of PI positive cells shows calcein AM at the working concentration of 2.67μM does not induce significantly more cell death than incubation in vehicle control in cells from the same donor (p = 0.25, paired sample analysis).

Uptake of PI for HCECs exposed to different concentrations of calcein AM was compared to that of unstained cells using flow cytometry. There was a correlation between calcein AM concentration and PI uptake, however, cell viability was not significantly different between unstained cells and those treated with 2.67μM calcein AM (2.72% vs 3.96%, p = 0.25, ANOVA) (Fig 4f). For ex-vivo corneas, the ECD, hexagonality ratio and the percentage of dead cells was not significantly between control and treatment corneas incubated with 2.67μM calcein AM(Fig 4a–4e).

### In-situ global imaging of graft viability using a porcine model

The porcine model replicated the steps of DMEK accurately and showed good face validity. Use of the anterior segment module supplied with Spectalis^™^ HRA allowed imaging of the entire graft at three time points (prior to graft preparation, following DMEK graft preparation and in-situ imaging post graft insertion). High quality images were obtained when imaging through either fresh or 24hr-old, swollen porcine corneas.

Fluorescent contrast between viable and non-viable areas was 15.41 (95% CI 8.78–22.0) with an average signal-to-noise ratio of 14.27 (95% CI 11.87–16.66). A receiver-operator-characteristic was plotted using mean fluorescence intensity from 50 viable and non-viable graft areas. Setting a fluorescent intensity of 40 units as a cut-off for thresholding viable are achieved a sensitivity of 96.6% with a specificity of 96.1% (Fig 2c)

### Degree of iatrogenic damage attributable to graft insertion and unfolding in DMEK

**Fig 5:**
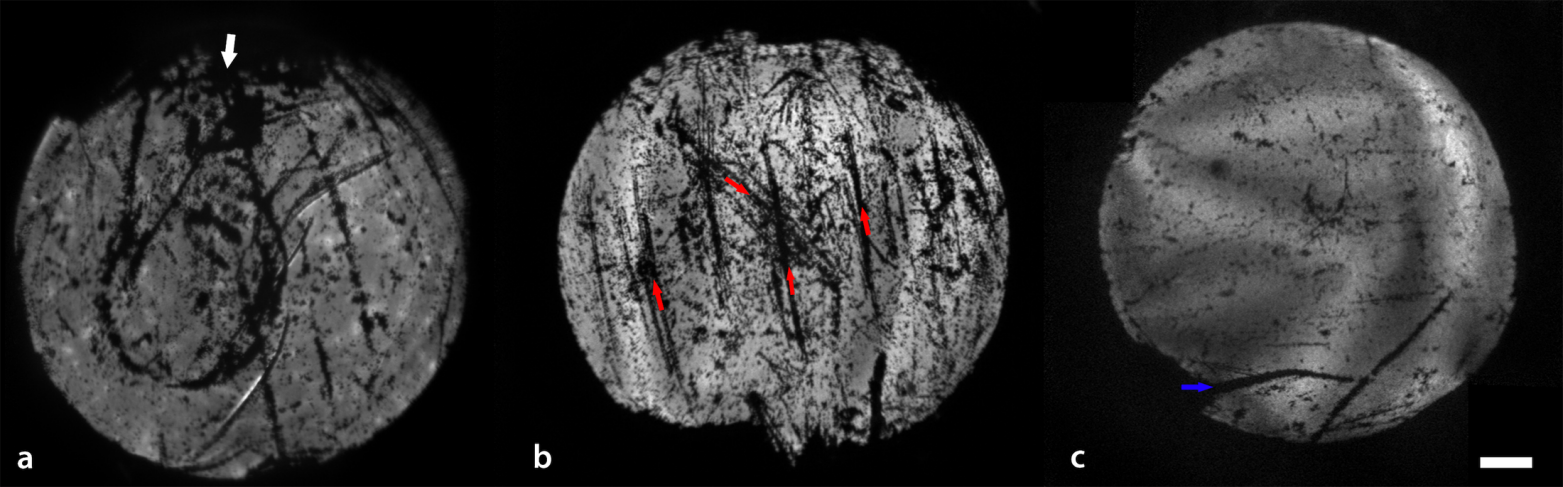
Global in-situ images of calcein AM stained DMEK grafts within the anterior chamber. (A) Areas of cell damage were seen at the portion of the graft that was incarcerated in the main wound during insertion (white arrow). (B) Multiple linear areas of cell loss (red arrows) are seen in a graft that was accidently ejected from the eye during insertion. The graft was still 73% viable after re-insertion and unfolding. (D) A tear in the graft originating from the orientation mark was noticed prior to insertion (blue arrow). This results in minimal cell death in the area adjacent to the tear and the cell in flap are largely viable. Scale bar 1mm.

Iatrogenic damage varied between 7–35% of the total graft area. Scenarios encountered clinically, such as tissue being incarcerated inside the main incision/paracentesis during insertion (n = 3) (Fig 5a), tissue being ejected from the eye (n = 1)(Fig 5b) and use of torn tissue (n = 2) (Fig 5c), were experienced in the pig eye surgical model. Whilst only seen in a small number of cases, each of these events was associated with a distinctive patterns of cell loss. Incarceration of tissue during insertion or unfolding resulted in loss of cells in that region. Multiple linear areas of cell loss were seen on the tissue ejected from the eye. Both of these patterns are consistent with physical trauma to the endothelial cells that lie on the outside of the tissue scroll. In two cases in which torn tissue was inserted, there was no significant cell loss in the areas adjacent to the tear or the flap.

### Comparison between in-situ and conventional in-vitro viability assessment methods

**Fig 6:**
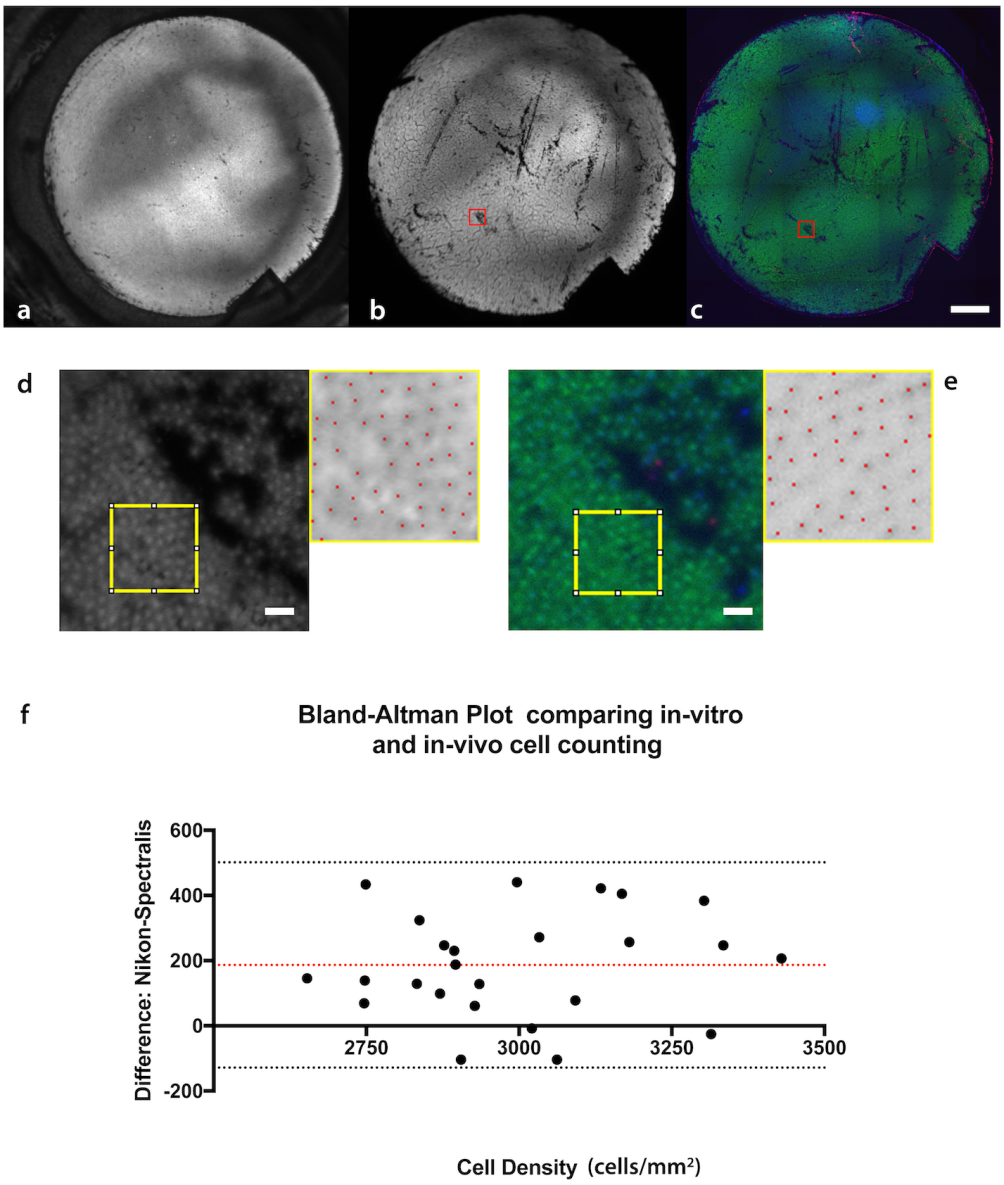
Comparison of viability assessments of in-situ and conventional in-vitro methods. (A) DMEK graft imaged post tissue preparation using the Spectralis^™^ HRA whilst in the Optisol viewing chamber. (B) DMEK tissue imaged in-situ after unfolding in the anterior chamber. (C) The same tissue was carefully removed from the anterior chamber after excising the cornea, re-stained, mounted in a customized, curved viewing chamber and reimaged. (D) High magnification images from corresponding areas (red squares) from in-situ and (E) in-vitro images were used to measure cell density (Scale bar 100μm). (F) A Bland-Altman plot was constructed to assess agreement between the two methods.

Areas of non-viability detected using the in-situ imaging showed good agreement with our global, in-vitro viability method (grafts had been re-incubated with Calcein AM and Ethidium Homodimer to confirm the loss of fluorescence accurately represented iatrogenic cell damage) (Fig 6a–6c).

Higher magnification imaging allowed visualization of individual cells (Fig 6d and 6e). Estimates of cell density based on calcein AM fluorescence showed a strong, positive correlation with counting Hoechst positive cells from the same graft region ex-vivo (R = 0.74, p<0.0001). There was a bias when counting cells based on in-situ calcein AM fluorescence; with this method detecting on average 187 fewer cells per mm^2^ (95%CI 120–250 cells/mm^2^) (Fig 6f).

**Fig 7:**
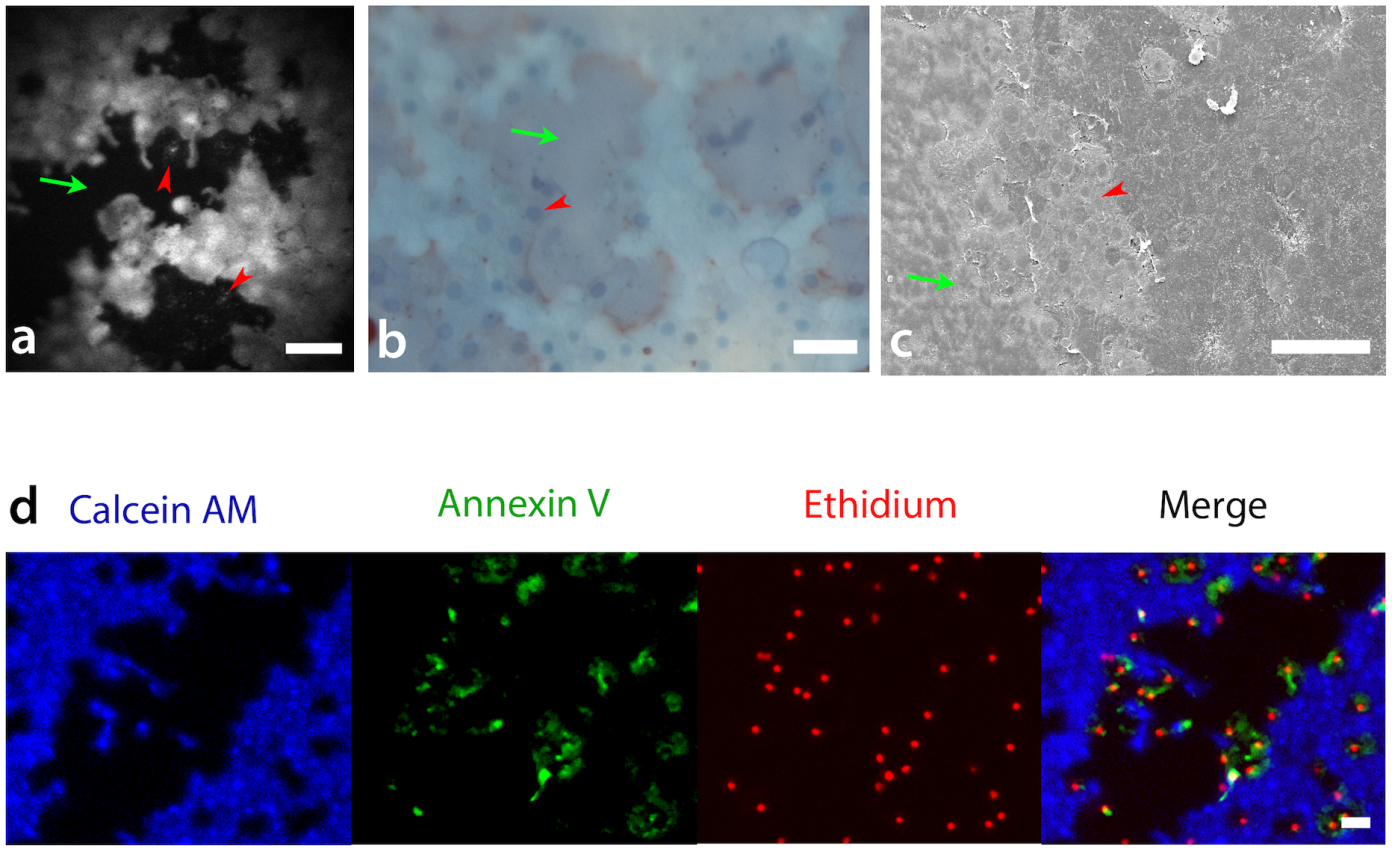
High magnification in-vivo, electron-microscopy and immuno-fluorescence live/dead imaging. (A) High magnification in-vivo images were taken using the Rostock corneal module. Individual cells and cell nuclei are clearly visible. Dead cells still attached to Descemet membrane (DM) (red arrow head) and bare areas of DM (green arrow can be seen), Scale bar 50μm. (B) The same patterns of cell loss (i.e. bare DM surrounded by dead cells) can be seen on trypan blue/alizarin red (Scale bar 50μm) viability staining and (C) scanning electron microscopy (100μm). (D) DMEK grafts triple stained with calcein AM blue, annexin V and ethidium show no overlap between calcein AM and early or late markers or apoptosis. Scale bar 100μm.

In addition, we compared in-vivo viability imaging to three other viability assessment methods: trypan blue/alizarin red staining, scanning electron microscopy and annexin V fluorescence. All methods showed the same patterns of cell loss; areas of bare Descemet membrane surrounded by attached but non-viable cells (trypan blue or ethidium homodimer positive) (Fig 7). In-situ, these non-viable cells could easily be seen at higher magnifications, in particular with use of the Rostock module (Fig 7a). Attached, dead cells had significantly diminished fluorescence (only marginally higher than that of the bare DM) allowing for easy discrimination between non-viable and viable attached cells.

Triple staining with calcein AM blue (live cells), ethidium homodimer (late apoptotic and necrotic cell death) and FITC conjugated annexin V (detects early apoptosis) showed no overlap between calcein AM and either of the dead cell stains, indicating that endothelial cells in both early and late stages of cell death are calcein AM negative (Fig 7).

## Discussion

In this study we describe a method for in-situ endothelial viability assessment using a single, short incubation with a fluorescent viability dye and a widely available clinical imaging device. To our knowledge, this is the first time a method for global in-situ graft viability assessment following corneal transplantation has been described. The method shows a very high sensitivity and specificity allowing easy segmentation of viable and non-viable areas of the graft at both the global and individual cell level. This detailed level of information is comparable to more time consuming in-vitro methods that required the tissue to be disposed of following staining eg. alizarin red/trypan blue dual staining or scanning electron microscopy. In-situ calcein AM imaging does not miss cells in the early stages of apoptotic cell death, suggesting it may have an advantage over other non-toxic viability dyes[28].

In current clinical practice, endothelial cell density is measured using specular or bright field microscopy of tissue prior to transplantation and specular or confocal microscopy in patients following transplantation. These measurements are prone to sampling errors, as cell density is not uniform across the cornea, increasing from the corneal apex to its periphery[29], and localized cell density can vary by as much as 10% within the central cornea alone[30]. Additionally, there is an assumption that all cells seen are viable. Inferences about the viability of the entire graft, made from cell density measurements taken from small, central areas of confluent cells, can be inaccurate[19] and falsely elevate the cell loss attributed to surgery itself[21].

Calcein AM requires two conditions in order to positively label cells. Firstly, the cells must be metabolically active for their cytoplasmic esterases to cleave calcein AM into fluorescent calcein. Secondly, in order for the polar calcein molecule to be retained within the cytoplasm, the cell membrane must remain intact. Iatrogenic damage occurring during the grafting process results in endothelial cell membrane compromise[18]. Our findings suggest this is sufficient to allow rapid escape of calcein through new cell membrane perforations, resulting in a loss of fluorescence and allowing new areas of non-viable cells to be identified at multiple time-points after the initial incubation, without the need for re-staining.

Whilst descriptions of cell loss during DSAEK insertion have been evaluated and used to modify insertion methods[16,31,32], to our knowledge, no descriptions of the patterns of cell loss occurring during DMEK tissue unfolding have been reported. Our findings suggest events a surgeon may associate with significant cell loss, such as unfolding torn tissue, may not significantly impact graft viability, and may not be automatic indications to discard tissue. Conversely, brief incarceration of tissue within a paracentesis, a not infrequent occurrence for those learning DMEK, resulted in significant cell damage in our limited study. Owing to the small samples size, further evaluation of the impact of specific surgical maneuvers is necessary to draw definitive conclusions.

The porcine eye model has been used to assess the feasibility of transplanting tissue engineered corneal grafts[33] and has long been used as a training model for corneal transplantation including DMEK[34]. The model has good face validity. Adding in-situ viability assessment to this model provides a good surrogate for animal surgery and will allow new modalities of EK and insertion devices to be assessed in a more realistic manner prior to clinical use.

Our method allows single cell viability can be determined in-vivo by the addition of contact or non-contact objective lenses to the confocal laser ophthalmoscope. At present this cannot be performed across the entire graft but approximates of global viability can be generated by combining information about macroscopic areas of cell death/loss with density and viability information taken from multiple high power fields[19], thereby partly compensating for the known variations in cell density across the graft.

In most samples, brighter nuclear staining with calcein AM was observed allowing these maxima to be used for semi-automate cell density assessment. This method seemed to systematically under-estimated cell density, with a bias of approximately 200 cells/mm^2^. However, all measurements fell between the 95% limits of agreement and therefore, with appropriate calibration, this method should give an accurate estimate of regional cell density. We noticed some pincushion distortion caused by the customized in-vivo optical set-up and this would account for lower cell density measurements. It should be possible to correct this with appropriate adaption of the imaging system.

We did not observe significant toxic effects for calcein AM used at a dose of 2.67μM in-vitro or ex-vivo and have begun use of calcein AM staining as part of our quality control process in animal models of EK. Grafts have cleared successfully without any observed differences between labeled and unlabeled controls, indicating calcein AM staining is not toxic in-vivo (data not shown). As work on tissue engineered grafts and cell injection therapy continues, a method of in-vivo viability assessment and cell imaging will be valuable[35]. Immediate in-vivo viability imaging will allow researchers to determine if graft failure is a consequence of iatrogenic cell loss during surgery or deficiencies in the cells being transplanted.

Whilst calcein AM has an excellent ability to determine graft viability, the fact it remains unbound within the cytoplasm means it is released from cells relatively quickly and is, therefore, not suitable as a long-term tracker. This seems to be an active process as corneas stored at 4°C retain fluorescence for at least 14 days, whereas it is rapidly lost in those stored in ex-vivo culture at 37°C. Our in-vivo experience reflects this, with fluorescence from calcein AM not visible 24hrs after transplantation. However, the most important time point at which to determine viability is immediately after surgery, meaning loss of fluorescence does not detract from the use of calcein AM for this indication. Use of other covalently linked dyes may allow longer-term tracking but these would likely stain dead but attached cells giving false positive results.

Fluorescent imaging is widely adopted in ophthalmology, with the Spectralis^™^ HRA designed primarily for clinical fluorescein angiography. Recently the combination of this clinical device and fluorescent apoptosis markers has been proposed as a clinical tool to detect glaucoma and Parkinson’s disease[36,37]. Whilst it would be straight forward to use calcein AM staining clinically, its use is currently restricted. To our knowledge, there is no good manufacturing process grade calcein AM available. Should this become available it is conceivable that a single incubation of graft tissue with calcein AM would be sufficient to allow assessment of graft viability at every stage from harvesting to implantation. Further work looking at long term tracking of endothelial cells in-vivo, such as the use of quantum dots [38] or fluorescent probes [39], is warranted, with the possibility of multiplexing these with calcein AM.

In summary, we have established a method for sequential graft viability assessment that can be used at every stage of transplantation. The methodology utilizes currently available clinical imaging devices and with minor modifications allows global (whole graft) and cell level viability and density assessment in-vivo.
